# Data on the identity of non-canonical complexes formed from proteasome subunits in vivo

**DOI:** 10.1016/j.dib.2016.11.048

**Published:** 2016-11-22

**Authors:** Lindsay J. Hammack, Andrew R. Kusmierczyk

**Affiliations:** Department of Biology, Indiana University-Purdue University Indianapolis, 723 West Michigan Street, Indianapolis, IN 46202, United States

**Keywords:** Proteasome, Yeast, Protein complex, Mass-spectrometry, Proteomics

## Abstract

The dataset presented here represents analysis supplied by the local proteomics core facility on samples submitted to it in support of the article “Assembly of proteasome subunits into non-canonical complexes in vivo” Hammack and Kusmierczyk (2016) [1]. This article provides the detailed protein contents of gel slices, cut from non-denaturing polyacrylamide gels, containing distinct protein complexes visualized following gel staining. The identification of the protein contents of these complexes was carried out by liquid chromatography tandem mass-spectrometry (LC–MS/MS).

**Specifications Table**

**Table:** 

Subject area	*Biology*
More specific subject area	*Molecular biology*
Type of data	*Excel files, word document*
How data was acquired	*The local proteomics core facility provides fee-for-service protein identification analysis by liquid chromatography tandem mass-spectrometry.*
Data format	*Processed*
Experimental factors	*Purified protein complexes isolated from various yeast strains were separated by native PAGE and stained to identify individual bands.*
Experimental features	*Gel slices, representing individual bands identified on non-denaturing polyacrylamide gels, were cut out and submitted for protein identification.*
Data source location	*Department of Biology; Indiana University-Purdue University Indianapolis; Indianapolis, IN, USA*
Data accessibility	*The processed data in Excel format, as supplied by the core facility, is with this article. The mass-spectrometry raw data collected by the proteomics core facility remains with the facility and was not provided to the researcher as part of the fee-for-service analysis.*

**Value of the data**

• The data support the identification of novel protein complexes comprised of proteasome subunits.
• Novel protein complexes identified invite additional experiments to further characterize them.
• Data also identify proteins associated with these novel protein complexes.

## 1 Data

The data contained here (Tables 1–8) are from the Excel files provided to the authors by the local proteomics core facility as part of the fee-for-service analysis of samples submitted (see Appendix). Each Excel file corresponds to a band cut out of a non-denaturing polyacrylamide gel in the referenced article [1]. Each Excel file includes information to direct the reader to the appropriate band that was analyzed. The Excel files contain details of the proteins identified in each sample submitted to the core facility, including: the number and sequence of each identified peptide, any modifications of each peptide, spectral counts, and coverage, etc. The title of each Excel file is meant to direct the reader to the actual band that was analyzed in the referenced article [1]. Additional Excel files are found in Appendix.

## 2 Experimental design, materials and methods

Non-denaturing polyacrylamide gel electrophoresis was carried out as described [1]. Bands (gel slices) were excised and submitted for analysis to the Indiana University School of Medicine Proteomics Core Facility. The facility carried out protein identification and provided processed data as Excel files. The Excel files were accompanied by a copy of a Word document that outlined the experimental procedure carried out by the core facility, and the subsequent data analysis, to identify the proteins within the sample. A copy of this word document is included in the Appendix.

## Figures and Tables

**Table 1:** Protein content of Band 1.

Accession	Description	Score	Coverage	# Unique Peptides	# Peptides	# PSMs
444302411	Chain Q, Yeast 20s Proteasome In Complex With The Vinyl Sulfone Lu112	236.09	78.74	3	16	90
323307097	Pre6p [Saccharomyces cerevisiae FostersO]	228.60	79.37	1	16	114
93279388	Chain U, Crystal Structure Of The Yeast 20s Proteasome In Complex With Bortezomib	201.77	88.07	22	22	78
323337524	Pre9p [Saccharomyces cerevisiae Vin13]	197.22	73.64	19	19	58
93279386	Chain S, Crystal Structure Of The Yeast 20s Proteasome In Complex With Bortezomib	183.95	83.69	19	19	60
256274381	Pba1p [Saccharomyces cerevisiae JAY291]	153.63	61.96	12	12	54
93279382	Chain O, Crystal Structure Of The Yeast 20s Proteasome In Complex With Bortezomib	152.91	52.00	12	12	65
178847521	Chain T, Yeast 20s Proteasome:glidobactin A-Complex	142.44	60.98	16	16	49
323309416	Pup3p [Saccharomyces cerevisiae FostersO]	135.74	46.60	8	8	38
444302412	Chain R, Yeast 20s Proteasome In Complex With The Vinyl Sulfone Lu112	109.18	59.62	17	17	34
403071955	Chain 2, Near-Atomic Resolution Structural Model Of The Yeast 26s Proteasome	103.01	31.80	9	9	69
151941458	conserved protein [Saccharomyces cerevisiae YJM789]	72.71	31.84	9	9	27
151944720	22.6 kDa proteasome subunit [Saccharomyces cerevisiae YJM789]	53.84	47.47	10	10	20
93279393	Chain Z, Crystal Structure Of The Yeast 20s Proteasome In Complex With Bortezomib	31.84	35.14	5	5	12
323305986	Ump1p [Saccharomyces cerevisiae FostersB]	31.83	22.97	3	3	22
207340308	YPR103Wp-like protein [Saccharomyces cerevisiae AWRI1631]	29.60	33.10	8	8	9
323337162	Pfk26p [Saccharomyces cerevisiae Vin13]	28.55	13.68	9	9	9
207345674	YFL007Wp-like protein [Saccharomyces cerevisiae AWRI1631]	26.43	4.62	6	6	8
741845	peptidyl-Glu protease	23.26	34.72	6	6	7
259146995	Ecm29p [Saccharomyces cerevisiae EC1118]	15.98	4.13	5	5	5
736313	unknown [Saccharomyces cerevisiae]	14.84	10.44	5	5	5
226279	mitochondrial assembly factor	11.90	7.17	3	3	4
323304344	Tdh2p [Saccharomyces cerevisiae FostersB]	11.63	20.98	3	3	4

This table pertains to Band 1 from Fig. 2 in the referenced manuscript.

**Table 2:** Protein content of Band 2.

Accession	Description	Score	Coverage	# Unique Peptides	# Peptides	# PSMs
444302411	Chain Q, Yeast 20s Proteasome In Complex With The Vinyl Sulfone Lu112	206.36	77.56	2	13	113
323307097	Pre6p [Saccharomyces cerevisiae FostersO]	182.00	78.17	1	13	82
323337524	Pre9p [Saccharomyces cerevisiae Vin13]	144.66	77.27	18	18	43
93279388	Chain U, Crystal Structure Of The Yeast 20s Proteasome In Complex With Bortezomib	141.43	83.13	19	19	57
323309416	Pup3p [Saccharomyces cerevisiae FostersO]	96.26	46.60	8	8	27
151946074	proteasome component Y7 [Saccharomyces cerevisiae YJM789]	94.51	37.20	11	11	40
403071955	Chain 2, Near-Atomic Resolution Structural Model Of The Yeast 26s Proteasome	67.94	31.03	7	7	41
172136	6-phosphofructo-2-kinase [Saccharomyces cerevisiae]	43.50	18.62	12	12	13
151944720	22.6 kDa proteasome subunit [Saccharomyces cerevisiae YJM789]	40.65	46.46	7	7	15
349577910	K7_Blm10p [Saccharomyces cerevisiae Kyokai no. 7]	39.81	6.35	11	11	13
259148570	Erg6p [Saccharomyces cerevisiae EC1118]	28.84	23.76	6	6	8
256270485	Pdc1p [Saccharomyces cerevisiae JAY291]	27.82	20.89	6	6	11
171457	enolase [Saccharomyces cerevisiae]	26.17	20.82	6	6	7
323336942	Ssc1p [Saccharomyces cerevisiae Vin13]	24.65	12.64	6	6	8
741845	peptidyl-Glu protease	24.17	36.27	6	6	8
259149737	Rpn8p [Saccharomyces cerevisiae EC1118]	24.00	16.57	4	4	7
323303909	Acs2p [Saccharomyces cerevisiae FostersB]	21.18	9.15	4	4	8
312258	PUP2 [Saccharomyces cerevisiae]	18.90	29.23	6	6	6
323355426	Rpn3p [Saccharomyces cerevisiae VL3]	18.64	15.48	5	5	6
223142	dehydrogenase isozyme I,alcohol	15.41	15.77	4	4	5
323352105	Eft2p [Saccharomyces cerevisiae VL3]	15.16	7.87	4	4	5
1103917	D-arabinono-1,4-lactone oxidase [Saccharomyces cerevisiae]	14.25	11.41	5	5	5
323333957	Rpn9p [Saccharomyces cerevisiae AWRI796]	13.22	13.80	3	3	4
323305986	Ump1p [Saccharomyces cerevisiae FostersB]	12.80	15.54	2	2	8

This table pertains to Band 2 from Fig. 2 in the referenced manuscript.

**Table 3:** Protein content of Band 3.

Accession	Description	Score	Coverage	# Unique Peptides	# Peptides	# PSMs

444302411	Chain Q, Yeast 20s Proteasome In Complex With The Vinyl Sulfone Lu112	625.80	67.72	2	16	313
323307097	Pre6p [Saccharomyces cerevisiae FostersO]	575.74	68.25	3	16	277
298508455	Chain U, Proteasome Activator Complex	487.69	67.40	2	14	174
93279388	Chain U, Crystal Structure Of The Yeast 20s Proteasome In Complex With Bortezomib	188.85	89.71	22	22	75
93279386	Chain S, Crystal Structure Of The Yeast 20s Proteasome In Complex With Bortezomib	119.42	78.11	14	14	37
323337524	Pre9p [Saccharomyces cerevisiae Vin13]	119.35	77.27	18	18	34
93279382	Chain O, Crystal Structure Of The Yeast 20s Proteasome In Complex With Bortezomib	117.98	52.00	11	11	48
323309416	Pup3p [Saccharomyces cerevisiae FostersO]	92.81	41.88	9	9	29
390980863	Chain T, Structure Of Yeast 20s Open-Gate Proteasome With Compound 34	90.73	51.65	9	9	27
403071955	Chain 2, Near-Atomic Resolution Structural Model Of The Yeast 26s Proteasome	72.80	46.74	7	7	47
256274381	Pba1p [Saccharomyces cerevisiae JAY291]	70.73	57.25	11	11	19
312258	PUP2 [Saccharomyces cerevisiae]	58.77	44.23	13	13	15
151944720	22.6 kDa proteasome subunit [Saccharomyces cerevisiae YJM789]	45.95	47.47	8	8	14
323305986	Ump1p [Saccharomyces cerevisiae FostersB]	35.64	41.89	5	5	20
323337162	Pfk26p [Saccharomyces cerevisiae Vin13]	35.29	16.54	10	10	10
93279395	Chain 2, Crystal Structure Of The Yeast 20s Proteasome In Complex With Bortezomib	34.81	65.31	8	8	12
93279393	Chain Z, Crystal Structure Of The Yeast 20s Proteasome In Complex With Bortezomib	32.74	35.59	6	6	11
151941458	conserved protein [Saccharomyces cerevisiae YJM789]	31.70	31.46	7	7	10
207340308	YPR103Wp-like protein [Saccharomyces cerevisiae AWRI1631]	30.22	25.09	6	6	10
93279394	Chain 1, Crystal Structure Of The Yeast 20s Proteasome In Complex With Bortezomib	23.31	27.04	3	3	7
226279	mitochondrial assembly factor	19.36	15.03	6	6	6
190407817	conserved hypothetical protein [Saccharomyces cerevisiae RM11-1a]	15.53	35.81	3	3	5
166007292	Chain E, Crystal Structure Of A Novel Chaperone Complex For Yeast 20s Proteasome Assembly	13.50	36.24	3	3	4
207345674	YFL007Wp-like protein [Saccharomyces cerevisiae AWRI1631]	12.97	2.78	4	4	4

This table pertains to Band 3 from Fig. 2 in the referenced manuscript.

**Table 4:** Protein content of Band 4.

Accession	Description	Score	Coverage	# Unique Peptides	# Peptides	# PSMs
444302411	Chain Q, Yeast 20s Proteasome In Complex With The Vinyl Sulfone Lu112	592.98	66.14	2	17	354
323307097	Pre6p [Saccharomyces cerevisiae FostersO]	538.85	66.67	2	17	281
93279388	Chain U, Crystal Structure Of The Yeast 20s Proteasome In Complex With Bortezomib	504.52	92.59	30	30	198
298508455	Chain U, Proteasome Activator Complex	417.71	65.64	2	15	145
323348418	Pre9p [Saccharomyces cerevisiae Lalvin QA23]	268.96	91.09	22	22	84
178847521	Chain T, Yeast 20s Proteasome:glidobactin A-Complex	214.58	62.72	14	14	72
93279382	Chain O, Crystal Structure Of The Yeast 20s Proteasome In Complex With Bortezomib	197.26	52.00	12	12	70
403071955	Chain 2, Near-Atomic Resolution Structural Model Of The Yeast 26s Proteasome	184.91	58.62	13	13	85
323309416	Pup3p [Saccharomyces cerevisiae FostersO]	176.76	42.93	8	8	49
93279386	Chain S, Crystal Structure Of The Yeast 20s Proteasome In Complex With Bortezomib	166.90	82.83	17	17	57
151944720	22.6 kDa proteasome subunit [Saccharomyces cerevisiae YJM789]	115.04	43.94	9	9	48
256274381	Pba1p [Saccharomyces cerevisiae JAY291]	92.42	57.25	11	11	28
312258	PUP2 [Saccharomyces cerevisiae]	86.96	61.54	17	17	25
323305986	Ump1p [Saccharomyces cerevisiae FostersB]	70.97	45.95	5	5	51
349577910	K7_Blm10p [Saccharomyces cerevisiae Kyokai no. 7]	53.82	7.89	13	13	17
166007287	Chain B, Crystal Structure Of A Novel Chaperone Complex For Yeast 20s Proteasome Assembly	51.61	76.54	9	9	12
123624	RecName: Full=Heat shock protein SSA2	45.22	27.70	3	11	13
323306163	Ssa1p [Saccharomyces cerevisiae FostersB]	41.81	24.18	1	10	12
151941458	conserved protein [Saccharomyces cerevisiae YJM789]	38.90	28.09	6	6	13
171541	glyceraldehyde-3-phosphate dehydrogenase (G3PD) [Saccharomyces cerevisiae]	35.39	33.73	6	6	11
323337162	Pfk26p [Saccharomyces cerevisiae Vin13]	35.23	16.54	10	10	10
190407817	conserved hypothetical protein [Saccharomyces cerevisiae RM11-1a]	29.15	37.16	5	5	10
190408474	conserved hypothetical protein [Saccharomyces cerevisiae RM11-1a]	25.09	5.06	6	6	8
323334738	Pre7p [Saccharomyces cerevisiae AWRI796]	24.16	18.48	2	2	6
3114294	Chain Z, Crystal Structure Of The 20s Proteasome From Yeast At 2.4 Angstroms Resolution	16.06	25.00	5	5	5
121575	RecName: Full=78 kDa glucose-regulated protein homolog; Short=GRP-78; AltName: Full=Immunoglobulin heavy chain-binding protein homolog; Short=BiP; Flags: Precursor	13.13	7.62	3	4	4

This table pertains to Band 4 from Fig. 2 in the referenced manuscript.

**Table 5:** Protein content of Band 5.

Accession	Description	Score	Coverage	# Unique Peptides	# Peptides	# PSMs
444302411	Chain Q, Yeast 20s Proteasome In Complex With The Vinyl Sulfone Lu112	1411.77	69.69	2	19	724
323307097	Pre6p [Saccharomyces cerevisiae FostersO]	1244.29	70.24	3	19	546
298508455	Chain U, Proteasome Activator Complex	1056.66	69.60	2	17	344
323352352	Pre6p [Saccharomyces cerevisiae VL3]	771.05	65.24	1	16	235
93279388	Chain U, Crystal Structure Of The Yeast 20s Proteasome In Complex With Bortezomib	114.49	79.01	17	17	47
323309416	Pup3p [Saccharomyces cerevisiae FostersO]	82.42	51.83	10	10	24
151944720	22.6 kDa proteasome subunit [Saccharomyces cerevisiae YJM789]	80.43	47.47	10	10	27
123624	RecName: Full=Heat shock protein SSA2	79.71	34.59	5	16	26
323306163	Ssa1p [Saccharomyces cerevisiae FostersB]	78.44	30.93	2	14	24
984187	transcription factor [Saccharomyces cerevisiae]	65.14	32.41	12	12	27
312258	PUP2 [Saccharomyces cerevisiae]	62.46	58.08	14	14	20
93279393	Chain Z, Crystal Structure Of The Yeast 20s Proteasome In Complex With Bortezomib	43.29	31.08	5	5	14
93279386	Chain S, Crystal Structure Of The Yeast 20s Proteasome In Complex With Bortezomib	41.02	60.94	10	10	12
93279382	Chain O, Crystal Structure Of The Yeast 20s Proteasome In Complex With Bortezomib	35.16	44.00	7	7	10
93279395	Chain 2, Crystal Structure Of The Yeast 20s Proteasome In Complex With Bortezomib	34.28	39.80	8	8	11
390980863	Chain T, Structure Of Yeast 20s Open-Gate Proteasome With Compound 34	24.33	31.40	6	6	10
121575	RecName: Full=78 kDa glucose-regulated protein homolog; Short=GRP-78; AltName: Full=Immunoglobulin heavy chain-binding protein homolog; Short=BiP; Flags: Precursor	24.28	11.44	5	6	8
93279392	Chain Y, Crystal Structure Of The Yeast 20s Proteasome In Complex With Bortezomib	19.31	25.47	4	4	6
403071955	Chain 2, Near-Atomic Resolution Structural Model Of The Yeast 26s Proteasome	18.70	21.46	3	3	10
151941041	proteasome biogenesis-associated [Saccharomyces cerevisiae YJM789]	17.59	11.59	3	3	6
93279394	Chain 1, Crystal Structure Of The Yeast 20s Proteasome In Complex With Bortezomib	16.80	6.44	1	1	5
323353866	Ubi4p [Saccharomyces cerevisiae VL3]	15.90	54.47	3	3	5

This table pertains to Band 5 from Fig. 2 in the referenced manuscript.

**Table 6:** Protein content of Band 6.

Accession	Description	Score	Coverage	# Unique Peptides	# Peptides	# PSMs
444302411	Chain Q, Yeast 20s Proteasome In Complex With The Vinyl Sulfone Lu112	1382.37	73.23	2	21	763
323307097	Pre6p [Saccharomyces cerevisiae FostersO]	1239.14	73.81	3	20	651
298508455	Chain U, Proteasome Activator Complex	1003.49	73.57	2	19	352
323352352	Pre6p [Saccharomyces cerevisiae VL3]	722.91	69.05	1	17	221
417149	RecName: Full=Heat shock protein SSA1; AltName: Full=Heat shock protein YG100	201.34	59.03	8	34	61
123624	RecName: Full=Heat shock protein SSA2	194.83	55.40	5	32	58
93279388	Chain U, Crystal Structure Of The Yeast 20s Proteasome In Complex With Bortezomib	160.69	79.42	20	20	63
151944720	22.6 kDa proteasome subunit [Saccharomyces cerevisiae YJM789]	131.89	47.47	9	9	49
323309416	Pup3p [Saccharomyces cerevisiae FostersO]	105.70	46.60	8	8	31
984187	transcription factor [Saccharomyces cerevisiae]	98.23	50.64	21	21	30
312258	PUP2 [Saccharomyces cerevisiae]	84.62	56.54	14	14	26
121575	RecName: Full=78 kDa glucose-regulated protein homolog; Short=GRP-78; AltName: Full=Immunoglobulin heavy chain-binding protein homolog; Short=BiP; Flags: Precursor	83.13	29.62	14	15	22
256274381	Pba1p [Saccharomyces cerevisiae JAY291]	71.41	45.65	8	8	19
390980834	Chain S, Structure Of Yeast 20s Open-Gate Proteasome With Compound 20	50.61	62.66	10	10	16
93279393	Chain Z, Crystal Structure Of The Yeast 20s Proteasome In Complex With Bortezomib	47.35	39.64	6	6	18
93279382	Chain O, Crystal Structure Of The Yeast 20s Proteasome In Complex With Bortezomib	37.34	44.00	8	8	13
403071955	Chain 2, Near-Atomic Resolution Structural Model Of The Yeast 26s Proteasome	33.68	37.16	7	7	13
390980863	Chain T, Structure Of Yeast 20s Open-Gate Proteasome With Compound 34	32.65	31.40	6	6	10
93279395	Chain 2, Crystal Structure Of The Yeast 20s Proteasome In Complex With Bortezomib	31.38	59.18	8	8	10
171457	enolase [Saccharomyces cerevisiae]	19.63	16.25	5	5	6
93279392	Chain Y, Crystal Structure Of The Yeast 20s Proteasome In Complex With Bortezomib	18.74	25.47	5	5	6
166007292	Chain E, Crystal Structure Of A Novel Chaperone Complex For Yeast 20s Proteasome Assembly	18.72	44.97	4	4	5
323353866	Ubi4p [Saccharomyces cerevisiae VL3]	13.64	54.47	3	3	4
256270485	Pdc1p [Saccharomyces cerevisiae JAY291]	11.03	9.39	2	2	3
171541	glyceraldehyde-3-phosphate dehydrogenase (G3PD) [Saccharomyces cerevisiae]	10.12	17.17	3	3	6

This table pertains to Band 6 from Fig. 2 in the referenced manuscript.

**Table 7:** Protein content of Band 7.

Accession	Description	Score	Coverage	# Unique Peptides	# Peptides	# PSMs
444302411	Chain Q, Yeast 20s Proteasome In Complex With The Vinyl Sulfone Lu112	402.40	62.99	3	15	176
323307097	Pre6p [Saccharomyces cerevisiae FostersO]	369.19	63.49	1	15	200
323337524	Pre9p [Saccharomyces cerevisiae Vin13]	15.59	9.09	1	1	4
323309416	Pup3p [Saccharomyces cerevisiae FostersO]	14.74	24.08	3	3	4
340707865	Chain A, Crystal Structure Of Yeast Hsp70 (BipKAR2) ATPASE DOMAIN	12.47	12.31	4	4	4
390980863	Chain T, Structure Of Yeast 20s Open-Gate Proteasome With Compound 34	12.10	14.88	3	3	4

This table pertains to Band 7 from Fig. 3 in the referenced manuscript.

**Table 8:** Protein content of Band 8.

Accession	Description	Score	Coverage	# Unique Peptides	# Peptides	# PSMs
444302411	Chain Q, Yeast 20s Proteasome In Complex With The Vinyl Sulfone Lu112	612.56	55.12	2	12	323
323307097	Pre6p [Saccharomyces cerevisiae FostersO]	537.41	55.56	2	12	334
298508455	Chain U, Proteasome Activator Complex	417.38	53.30	2	10	130
417149	RecName: Full=Heat shock protein SSA1; AltName: Full=Heat shock protein YG100	96.44	38.01	5	17	26
123624	RecName: Full=Heat shock protein SSA2	92.78	33.18	3	16	24
256270485	Pdc1p [Saccharomyces cerevisiae JAY291]	30.17	16.67	5	5	8
93279388	Chain U, Crystal Structure Of The Yeast 20s Proteasome In Complex With Bortezomib	21.66	20.16	4	4	6
121575	RecName: Full=78 kDa glucose-regulated protein homolog; Short=GRP-78; AltName: Full=Immunoglobulin heavy chain-binding protein homolog; Short=BiP; Flags: Precursor	15.44	8.80	3	4	4
323333274	Fur1p [Saccharomyces cerevisiae AWRI796]	10.70	20.77	3	3	3

This table pertains to Band 8 from Fig. 3 in the referenced manuscript.

## Footnotes

Appendix A Supplementary data associated with this article can be found in the online version at http://dx.doi.org/10.1016/j.dib.2016.11.048.
