# Supplementary material for: Data on the identity of non-canonical complexes formed from proteasome subunits in vivo
Source: Data Brief. 2016 Nov 22;9:1130–7. doi: 10.1016/j.dib.2016.11.048 (PMC5128733; doi:10.1016/j.dib.2016.11.048)
Supplement: Supplementary file 2 — Supplementary material [file mmc2.zip › Core Facility Sample Analysis.docx]

- **Research Goal:** Protein identification from gel using LC-MS/MS.
- **Materials**: 10 gel samples
- **Sample Prep**:

The gel was detained, reduced with 10 mM DTT in 10 mM ammonium bicarbonate and then alkylated with 55 mM iodoacetamide (prepared in 10 mM ammonium bicarbonate). Alkylated samples were digested by trypsin (Promega) overnight at 37°C. Digested peptides were extracted from the gel spots with 1) 50% ACN /49.9%H_2_O/0.1% TFA; 2) 99.9% ACN/0.1% TFA.

- **Instrument**: Thermo-Fisher Scientific Orbitrap Velos Pro and Thermo Dionex UltiMate 3000 RSLC nanoflow system

Digested peptides were injected onto the C18 column. Peptides were eluted with a linear gradient from 3 to 35% acetonitrile (in water with 0.1% FA) developed over 70 min at room temperature at a flow rate of 300 nL/min, and effluent was electro-sprayed into the mass spectrometer. A blank was run prior to and between the sample runs to make sure there was no significant signal from solvents, column or carryovers.

**Columns:** Analytical column: PepMap®, C18, 2um, 100A, 75um×15cm

Trapping column: PepMap®, C18, 3um, 100A, 300um id ×5mm

- **Database Search:**

Database search was carried out using Sequest^TM^ algorithms against ***yeast_SC from Uniprot***

**Summary of Findings:** Please see attached files. The proteins are reported with high peptide confidence level (over 90%).

***It is our recommendation to ONLY consider proteins identified with at least 2 unique sequences as “confidently identified”. Proteins identified with only one peptide (or called “single-hit wonder”) should be viewed as questionable (Carr, S., Molecular & Cellular Proteomics 3.6, 2004, pp. 531-533).***
